# Self‐care training using the Tablo hemodialysis system

**DOI:** 10.1111/hdi.12890

**Published:** 2020-10-12

**Authors:** Troy J. Plumb, Luis Alvarez, Dennis L. Ross, Joseph J. Lee, Jeffrey G. Mulhern, Jeffrey L. Bell, Graham E. Abra, Sarah S. Prichard, Glenn M. Chertow, Michael A. Aragon

**Affiliations:** ^1^ University of Nebraska Nebraska Medical Center Omaha Nebraska 68198 USA; ^2^ Palo Alto Medical Foundation 795 El Camino Real, Palo Alto California 94301 USA; ^3^ Kansas Nephrology Research Institute 1007 N. Emporia, Wichita Kansas 67214 USA; ^4^ Nephrology Associates Medical Group 3660 Park Sierra #208, Riverside California 92505 USA; ^5^ Fresenius Kidney Care Pioneer Valley Dialysis 208 Ashley Ave West Springfield Massachusetts 01089 USA; ^6^ Southwest Georgia Nephrology Clinic 1200 North Jefferson Street, Albany Georgia 31701 USA; ^7^ Stanford University 300 Pasteur Drive, 1st floor, Suite A175, Stanford California 94305 USA; ^8^ Western University London Ontario Canada; ^9^ Stanford University School of Medicine 1070 Arastradero Road, Palo Alto California 94034 USA; ^10^ DaVita Grapevine at Home 1600 W. Northwest Hwy, Suite 100 Grapevine Texas 76051 USA

**Keywords:** Self‐care, hemodialysis, training

## Abstract

**Introduction:**

Recently published results of the investigational device exemption (IDE) trial using the Tablo hemodialysis system confirmed its safety and efficacy for home dialysis. This manuscript reports additional data from the Tablo IDE study on the training time required to be competent in self‐care, the degree of dependence on health care workers and caregivers after training was complete, and participants' assessment of the ease‐of‐use of Tablo.

**Methods:**

We collected data on the time required to set up concentrates and the Tablo cartridge prior to treatment initiation. We asked participants to rate system setup, treatment, and takedown on a Likert scale from 1 (very difficult) to 5 (very simple) and if they had required any assistance with any aspect of treatment over the prior 7 days. In a subgroup of 15 participants, we recorded the number of training sessions required to be deemed competent to do self‐care dialysis.

**Findings:**

Eighteen men and 10 women with a mean age of 52.6 years completed the study. Thirteen had previous self‐care experience using a different dialysis system. Mean set up times for the concentrates and cartridge were 1.1 and 10.0 minutes, respectively. Participants with or without previous self‐care experience had similar set‐up times. The mean ease‐of‐use score was 4.5 or higher on a scale from 1 to 5 during the in‐home phase. Sixty‐five percent required no assistance at home and on average required fewer than four training sessions to be competent in managing their treatments. Results were similar for participants with or without previous self‐care experience.

**Conclusions:**

Participants in the Tablo IDE trial were able to quickly learn and manage hemodialysis treatments in the home, found Tablo easy to use, and were generally independent in performing hemodialysis.

## INTRODUCTION

Conventional in‐center hemodialysis performed by health care professionals remains the most widely used dialysis modality worldwide. In the United States, in‐center hemodialysis currently accounts for over 85% of dialysis treatments.^1^ Self‐care dialysis, whether in the home or in center, empowers patients to take a more active role in their treatment. Although definitions of self‐care can vary, we believe self‐care to be defined as follows:

Dialysis performed with little or no professional assistance by a patient or caregiver who has completed an appropriate course of training and, at a minimum, sets up the equipment used in the treatment, touches the machine and responds to alarms, manages access site pre‐ and post‐treatment (with or without self‐cannulation) and takes and records body weight and vital signs.

The most common form of self‐care dialysis is self‐administered peritoneal or hemodialysis in the home.^1^ Observational studies of home dialysis have reported potential benefits in health‐related quality of life, various intermediate outcomes, and in some studies, survival, along with reduced overall cost of care.^2^, ^3^, ^4^, ^5^, ^6^ In New Zealand and Australia, community houses, which support self‐care hemodialysis, report mortality and health‐related quality of life outcomes similar to home hemodialysis.^7^, ^8^The recently introduced Advancing American Kidney Health (AAKH) initiative seeks to increase the use of home therapies with the goal of 80% of new patients with end‐stage kidney disease (ESKD) treated either with pre‐emptive kidney transplantation or with home dialysis by 2025.^9^

Despite the observed benefits of home dialysis and the increased focus and support expected from the AAKH, environmental and social barriers to home dialysis will continue to present a challenge for some patients willing to perform self‐care.^10^, ^11^, ^12^, ^13^ Single center observational studies of in‐center self‐care hemodialysis have reported positive findings with respect to hospitalization rates, missed treatments and survival compared to patients receiving conventional in‐center hemodialysis.^14^ Self‐care approaches have been successful in other chronic disease states as well as in chronic kidney disease.^15^, ^16^, ^17^, ^18^, ^19^, ^20^, ^21^, ^22^

One of the barriers to self‐care hemodialysis both in the home and in‐center settings has been the absence of technology that enables patients to easily learn and confidently manage their treatment in a time efficient manner with minimal burden on health care workers or caregivers.^15^ The Tablo hemodialysis system is an all‐in‐one, patient‐centric system designed to be easy to learn and manage with on‐demand dialysate production and two‐way wireless connectivity. A recent publication reported on the successful achievement of all safety and efficacy endpoints in the investigational device exemption (IDE) trial using Tablo for home hemodialysis.^23^ Previous publications have also demonstrated the ease‐of‐use of Tablo in human factors^24^ and clinical observational^25^ studies.

The purpose of this manuscript is to report data on elements of training from the Tablo IDE study including, the time required for patients to be competent in self‐care, the time required for patients to perform specific system‐related activities, ease‐of‐use and the degree of dependence on health care workers and caregivers once successfully trained.

## METHODS

The Tablo IDE study was a prospective, multicenter, open label, crossover trial describing patient experience and outcomes on in‐center and in‐home hemodialysis using the Tablo hemodialysis system. Participants remained in the trial for approximately 21 weeks during which time they were prescribed hemodialysis with Tablo four times per week. The trial consisted of four treatment periods during which Tablo was utilized: a Run‐in period of 1 week in‐center, 32 treatments (approximately 8 weeks) in‐center during which dialysis was managed by a health care professional, a 2–4 weeks transition period during which the patient and caregiver were trained on the system and 32 treatments (approximately 8 weeks) of in‐home self‐care hemodialysis. Thirty patients were enrolled in the trial and 28 completed all phases of the trial. The original study protocol and amendments were approved by the United States Food and Drug Administration and were registered on www.clinicaltrials.gov (NCT02460263). Details of the study design and the primary and secondary efficacy and safety outcomes were previously published.^23^

For the 28 participants who completed self‐care training during the transitional phase and proceeded to the in‐home phase of the study, we collected data on the time required to set up concentrates and the Tablo cartridge prior to treatment initiation. The recorded times were based on the time it took for a participant when prompted by the graphical user interface (GUI) to start a task until the screen for the next task was reached. These times were stored in the digital log file for each treatment that was either wirelessly transmitted or stored on a USB drive for future data extractions.

For the purpose of analysis, we stratified patients according to prior modality (previously on self‐care at home or conventional in‐center hemodialysis). To assess system ease‐of‐use for self‐care, we asked participants weekly to rate system setup, treatment, and takedown on a Likert scale from 1 (very difficult) to 5 (very simple) during the transition /training phase and the in‐home phase of the study. For the 13 participants previously performing self‐care hemodialysis, we asked them to recall the ease of use and their need for assistance on the previous dialysis system used.

We asked participants weekly during the in‐home phase of the study if they had required any assistance with any aspect of treatment over the prior 7 days and if so, with which of the following tasks: Machine Set Up, Clearing of Alarms, Machine Take Down, Fistula/Needling or Catheter Connection.

A protocol amendment allowed data to be collected for the final 15 enrolled participants in the study to determine the number of training sessions required for the participant to be deemed competent at setting up and managing Tablo. Patients were deemed capable of independent device operation when competency had been achieved with all tasks in the opinion of the training health care provider. Cannulation training data were not collected. We assessed data on the following four tasks: setup of Tablo, treatment management, treatment takedown and alarm management as well the overall number of training sessions required to be competent in managing Tablo for self‐care use.

For the 28 patients who completed self‐care training during the transition phase, we calculated the mean, minimum and maximum time for each step.

## RESULTS

Thirty patients were enrolled in the trial. During the Transition phase, one participant died due to cardiac arrest during the interdialytic period. This event was deemed unrelated to dialysis and unrelated to Tablo. One participant decided not to continue with the study and withdrew consent prior to entering the in‐home period. Twenty‐eight (97%) of 29 eligible participants from eight sites completed the 4‐week transition phase and 8‐week in‐home phase of the trial. Fifteen participants had the number of training sessions during the transition phase of the trial recorded. Five of these 15 participants had previous self‐care experience.

Table 1 shows demographic data for the 28 participants who completed the transition and in‐home phases of the trial and the subgroup of 15 who had their training sessions recorded.

**Table 1:** Demographics of the 28 patients who completed all four phases of the trial and the 15 who had training sessions measured

	Category	Completed trial (N = 28) Total (%)	Training sessions assessed (N = 15) Total (%)
Sex	Male	18 (64)	9 (60)
	Female	10 (36)	6 (40)
Age (years)	Mean	52.6	58.5
	SD	12	8.4
	Range	26–71	40–71
BMI (kg/m^2^)	Mean	32.2	32.0
	SD	4.9	4.8
	Range	23.6–40.5	23.6–40.0
Race/Ethnicity	White	16 (57)	8 (53)
	Hispanic or Latino	7 (25)	3 (20)
	Not Hispanic or Latino	9 (32)	5 (33)
	Black or African American	12 (43)	7 (47)
Access type	Fistula	21 (75)	11 (73)
	Catheter	4 (14)	2 (13)
	Graft	3 (11)	2 (13)
Cause of ESKD	Diabetes	13 (46)	11 (73)
	Hypertension	1 (4)	0 (0.0)
	Kidney transplant	3 (11)	0 (0.0)
	Polycystic kidney disease	2 (7)	1 (7)
	Glomerulonephritis	2 (7)	1 (7)
	Other	7 (25)	2 (13)
Previous in‐home experience		13 (46)	5 (33)

The number of observations and the average times required for setting up the concentrates and cartridge on Tablo during the home phase are shown in Table 2. The maximum number of observations for each participant is 32. We recorded an average of 28 observations per patient. We report the number of observations (n), the average time taken by each patient for the set‐up of Tablo concentrates and the cartridge as well as the minimum and maximum time for each step for each participant. There was a trend for the time required to perform these tasks to decrease between weeks one and four, but this did not reach statistical significance.

**Table 2:** Concentrate and cartridge setup times during the home treatment period: average (maximum and minimum) in minutes

Subjects previously on in‐center HD	N	Setup time for concentrates minutes (minimum‐maximum)	Set‐up time for cartridge minutes (minimum‐maximum)
1	29	0.7 (0.3–4.4)	8.8 (2.6–20.9)
2	32	0.6 (0.4–1.9)	11.3 (5.4–83.6)
3	1	0.50	1.90
4	32	0.4 (0.3–0.6)	4.3 (2.3–7.9)
5	32	0.9 (0.1–2.7)	8.3 (4.2–27.8)
6	21	1.1 (0.4–2.1)	8.0(4.9–11.9)
7	31	1.5 (0.5–4)	13.1 (5.3–28.6)
8	31	1.4 (0.4–4.7)	10.0 (6.1–14.8)
9	30	0.9 (0.5–2.1)	17.1 (5.3–76)
10	20	0.7 (0.3–1.7)	13.3 (4.4–43.8)
11	24	1.2 (0.4–7.9)	11.9 (5.6–36.7)
12	32	0.7 (0.3–1.4)	8.7 (4.8–60.7)
13	32	0.6 (0.2–1.9)	6.9 (2.4–12.1)
14	16	0.6 (0.4–1.4)	7.4 (5.0–10.0)
15	28	0.7 (0.4–1.6)	8.0 (4.9–20.4)
Average		0.93	9.35
Subjects previously on home HD			
1	22	2.0 (0.3–4.5)	16.5 (5–53.1)
2	30	0.9 (0.4–2.6)	5.4 (3.2–16.8)
3	29	1.4 (0.5–5.1)	8.4 (5.8–13.3)
4	31	1.5 (0.5–8.5)	31.5 (6.7–265.5)
5	31	0.6 (0.3–2.1)	6.8 (3.3–16)
6	32	0.4 (0.3–.9)	4.1 (2.8–5.5)
7	32	0.7 (0.4–1.6)	11.4 (3.4–188)
8	27	0.8 (0.2–4.5)	7.0 (3.1–12.8)
9	32	0.7 (0.5–1.3)	7.3 (3.7–14)
10	32	0.6 (0.3–3.2)	5.6 (2.4–8.7)
11	32	1.1 (0.3–10.8)	12.5 (2.1–69.4)
12	32	0.7 (0.3–4.4)	4.7 (2.5–12.2)
13	32	1.6 (0.3–3.6)	10.1 (2.2–18.5)
Average		1.22	10.28
			
Overall		1.09	10.02

N is the number of observations made for each patient.

Table 3 shows ease‐of‐use survey data with respect to set‐up, treatment and takedown. Self‐care naïve patients rated Tablo an average 4.3 out of 5 for each of setup, treatment, and takedown while training during the transition phase. These ratings increased to 4.6 for setup and treatment and 4.7 for takedown while performing self‐care during the 8‐week in‐home phase. Patients with previous self‐care hemodialysis experience rated Tablo 4.5 for set up during both phases of the study and treatment and takedown were rated 4.6 for both phases. Recall of the ease of use of their previous device is also shown.

**Table 3:** Ease of use ratings of previous machine by recall at baseline for patients with previous self‐care experience, during transition/training on Tablo and during in‐home period on Tablo

	Previous device	Transition/training period		In‐home period	
Ease of use	Subjects previously on self‐care Avg (Min/Max)	Prior self‐care Avg (Min/Max)	New to self‐care Avg (Min/Max)	Prior self‐care Avg (Min/Max)	New to self‐care Avg (Min/Max)
Setup	3.5 (1,5)	4.5 (3,5)	4.3 (3,5)	4.5 (4,5)	4.6 (1,5)
Treatment	3.3 (2,5)	4.6 (3,5)	4.3 (3,5)	4.6 (4,5)	4.6 (3,5)
Takedown	3.8 (1,5)	4.6 (3,5)	4.3 (2,5)	4.6 (4,5)	4.7 (3,5)

Likert scale: 1 very difficult, 2 somewhat difficult, 3 neutral, 4 somewhat simple, 5 very simple.

**Figure 1:**
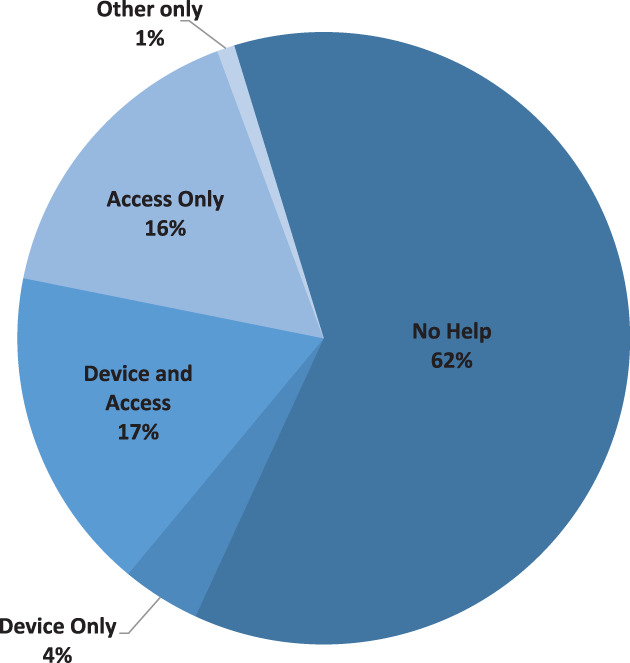
Assistance required by treatment week for all participants during the in‐home phase. [Color figure can be viewed at wileyonlinelibrary.com]

Figure 1 shows the assistance needed from a trained care partner for all participants during the home phase of the study. During the in‐home phase, participants completed a total of 224 patient‐weeks (28 patients × 8 weeks) of self‐care hemodialysis. Responses regarding need for assistance were obtained in 96% (216/224) of treatment weeks. Participants reported the ability to perform treatments on Tablo independently during 62% (134 of 216) of weeks.

**Figure 2:**
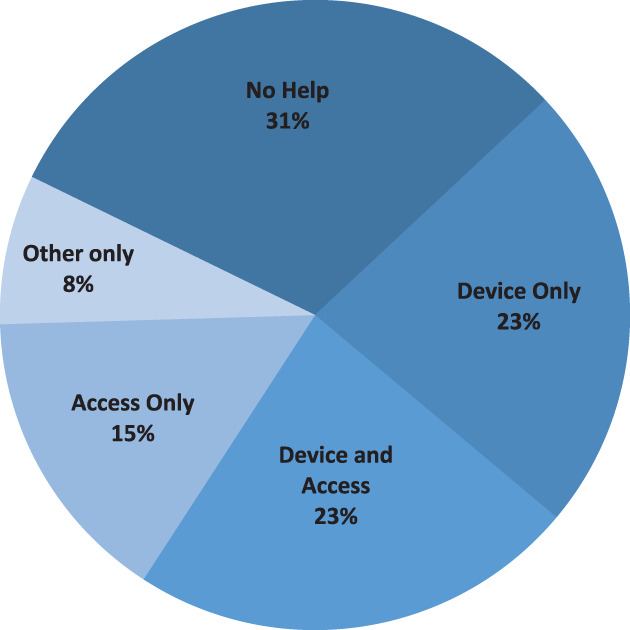
Percent of participants with previous self‐care experience needing treatment assistance at enrollment. [Color figure can be viewed at wileyonlinelibrary.com]

**Figure 3:**
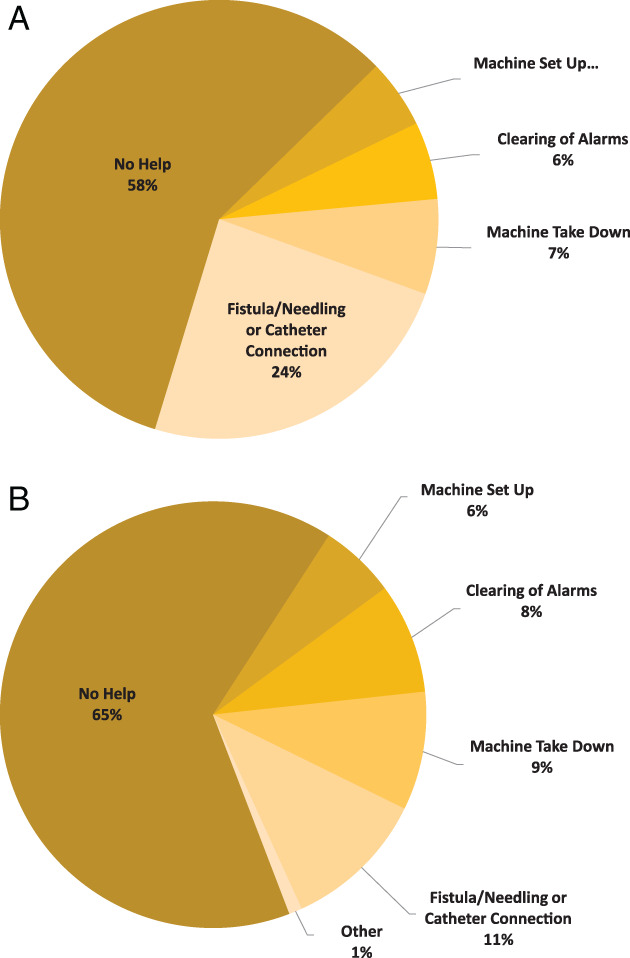
(a) Percent of weeks participants with previous self‐care experience required assistance using Tablo at home. All participants were prescribed four treatments per week. (b) Percent of weeks that participants new to self‐care required assistance using Tablo at home. All participants were prescribed four treatments per week. [Color figure can be viewed at wileyonlinelibrary.com]

Figures 2 and 3 show the need for assistance for participants with previous self‐care experience at baseline and during the in‐home phase and the need for assistance for those participants without previous self‐care experience during the in‐home phase. At baseline, nine (69%) participants with previous self‐care experience required some form of assistance. Three (23%) required assistance with device related steps only (setup, alarm management or takedown), three (23%) required assistance with device and dialysis access cannulation or connection, two (15%) with access cannulation or connection only and one (8%) reported needing assistance with other treatment related tasks. During the in‐home phase, prior self‐care participants completed 97 of a possible 104 (93%) weekly surveys regarding assistance. Forty‐one (42%) of treatment weeks required some assistance. Seventeen (18%) were for device related steps (setup, alarm management, or takedown) and 23 (24%) required assistance with dialysis access cannulation or connection.

During the in‐home phase, participants new to self‐care completed 119 of a possible 120 (99%) weekly surveys. Forty‐two (35%) surveys reported requiring some form of assistance, of which, 27 (23%) were for device related steps (setup, alarm management, or takedown).

The requirement for some form of assistance among participants with or without previous self‐care experience was not meaningfully different.

Table 4 shows the number of training sessions required to be considered competent by the training staff for each of 15 participants for whom these data were captured. The average number of training sessions required to achieve all of the steps successfully was 3.6 sessions for participants with previous self‐care dialysis experience and 3.9 sessions for those with only conventional in‐center experience.

**Table 4:** Training sessions required to achieve self‐care in 15‐participant sub cohort (setup, treatment, takedown, alarms, and overall competency)

Previously in‐center					
	Setup	Treatment	Takedown	Alarms	Overall competency
Participant ID					
1	2	2	2	2	2
2	2	1	1	6	6
3	2	3	2	5	5
4	2	1	1	1	2
5	1	1	1	2	2
6	5	5	5	9	9
7	1	1	2	1	2
8	4	6	6	6	6
9	1	1	2	1	2
10	2	2	3	2	3
Average	2.2	2.3	2.5	3.5	3.9
Previously in‐home					
11	3	3	3	3	3
12	2	2	2	2	2
13	2	2	3	2	3
14	5	5	5	2	5
15	5	5	5	5	5
Average	3.40	3.40	3.60	2.80	3.60
Average (all pts)	2.60	2.7	2.9	3.3	3.8

## DISCUSSION

In this study, a detailed analysis of training within the IDE trial evaluating Tablo in the home, participants were able to successfully and rapidly set up Tablo's concentrates and cartridge to start therapy. Accounting for Tablo's automated prime, which is approximately 8 minutes in duration, the estimated average time to initiate therapy could be as low as 20 minutes with further reduction possible with continued use of Tablo. It is noteworthy that participants with no prior self‐care experience did not require longer set up or training times on Tablo compared to those with self‐care hemodialysis experience. Whether previously familiar with self‐care or self‐care naïve, participants generally rated Tablo setup, treatment, and takedown simple to very simple initially and after several months of use. Participants in both groups demonstrated an ability to perform device‐related tasks with minimal and most often, no, assistance.

The growth of self‐care hemodialysis, whether in‐center or in‐home, will require elimination of a number of barriers. Better training of nephrologists, nephrology nurses and allied health care personnel, accommodation by dialysis providers for training and self‐care treatment, and reimbursement models that align to incentivize self‐care will be required to effectuate a successful transition to a self‐care hemodialysis modality and an empowered population of patients with ESKD.^11^ In‐center self‐care hemodialysis is an underutilized modality option that offers some of the advantages of home hemodialysis for patients who are unable to overcome barriers to home self‐care, including fear of cannulation, physical space limitation, care giver unavailability or patient preference.^10^, ^11^, ^12^, ^13^

Irrespective of previous self‐care hemodialysis experience, IDE trial participants required an average of fewer than four sessions to complete all steps required to operate Tablo, and to be considered competent in self‐care hemodialysis by experienced personnel.

The study has several strengths. Retention and adherence were high. While the sample size was small, participants were diverse by age, sex, race/ethnicity, primary cause of kidney disease, dialysis vintage, comorbidity, “home locale” (e.g., single‐family home, apartment, recreational vehicle) and experience with home hemodialysis therapy. However, the participants were all aware that this was a study and were motivated to complete the protocol. This may limit the generalizability of the results. Assessments of ease‐of‐use were collected repeatedly; as such, time‐averaged values during each study phase were more likely to reflect true patient experience and less prone to misclassification than in other research or real‐world settings. The study also has several limitations. The time to complete various set up steps was variable. Although we precisely calculated time based on a participant moving from one GUI screen to the next, we may have overestimated the time dedicated to machine set up if the participant had stepped away from Tablo or were distracted by a phone call or text message. With respect to the number of training sessions required for competency, we did not employ formal assessments of competency; thus, there was likely some center‐to‐center variability. In addition, although the patients received no formal training during the in‐center phase of the study, it is possible that they may have learned about certain aspects of dialysis with Tablo through observation of the health care team but this would not be inherently different with Tablo compared to other devices. All trainers were experienced home dialysis nurses who enabled all participants who entered the in‐home phase to be successful. As designed, all participants were treated with the Tablo hemodialysis system in all study phases. Thus, we could not determine whether there were differences in ease‐of‐use among Tablo and other home or conventional in‐center hemodialysis equipment.

In summary, this study confirms and extends previously published data on Tablo demonstrating that the novel hemodialysis system can be managed easily and successfully by a broad range of patients with a low requirement for assistance.^24^, ^25^ Prior studies demonstrate that Tablo delivers adequate clearance and accurate ultrafiltration rates thrice weekly.^23^ Its advanced data capabilities and on demand dialysate generation make it well suited for self‐care in health care facilities of any size or configuration (e.g., free standing dialysis units, hospitals, long‐term acute care, rehabilitation, and skilled nursing facilities) or at home. Moreover, the small footprint and on‐demand dialysate production allow for the development of new sites and models of care that could encourage and empower patients to take a more active role in their hemodialysis treatment.
